# GZ17-6.02 Interacts With [MEK1/2 and B-RAF Inhibitors] to Kill Melanoma Cells

**DOI:** 10.3389/fonc.2021.656453

**Published:** 2021-04-08

**Authors:** Laurence Booth, Cameron West, Daniel Von Hoff, John M. Kirkwood, Paul Dent

**Affiliations:** ^1^ Department of Biochemistry and Molecular Biology, Virginia Commonwealth University, Richmond, VA, United States; ^2^ Genzada Pharmaceuticals, Sterling, KS, United States; ^3^ Translational Genomics Research Institute (TGEN), Phoenix, AZ, United States; ^4^ Melanoma and Skin Cancer Program, Hillman Cancer Research Pavilion Laboratory, University of Pittsburgh Cancer Institute, Pittsburgh, PA, United States

**Keywords:** B-Raf mutation V600E, GZ17-6.02, autophagy, er stress, HDAC

## Abstract

We defined the lethal interaction between the novel therapeutic GZ17-6.02 and the standard of care combination of the MEK1/2 inhibitor trametinib and the B-RAF inhibitor dabrafenib in PDX isolates of cutaneous melanoma expressing a mutant B-RAF V600E protein. GZ17-6.02 interacted with trametinib/dabrafenib in an additive fashion to kill melanoma cells. Regardless of prior vemurafenib resistance, the drugs when combined interacted to prolong ATM S1981/AMPK T172 and eIF2α S51 phosphorylation and prolong the reduced phosphorylation of JAK2 Y1007, STAT3 Y705 and STAT5 Y694. In vemurafenib-resistant cells GZ17-6.02 caused a prolonged reduction in mTORC1 S2448, mTORC2 S2481 and ULK1 S757 phosphorylation; regardless of vemurafenib resistance, GZ17-6.02 caused a prolonged elevation in CD95 and FAS-L expression. Knock down of eIF2α, Beclin1, ATG5, ATM, AMPKα, CD95 or FADD significantly reduced the ability of GZ17-6.02 to kill as a single agent or when combined with the kinase inhibitors. Expression of activated mTOR, activated STAT3, activated MEK1 or activated AKT significantly reduced the ability of GZ17-6.02 to kill as a single agent or when combined with kinase inhibitors; protective effects that were significantly less pronounced in cells treated with trametinib/dabrafenib. Regardless of vemurafenib resistance, the drugs alone or in combination all reduced the expression of PD-L1 and increased the levels of MHCA, which was linked to degradation of multiple HDAC proteins. Our findings support the use of GZ17-6.02 in combination with trametinib/dabrafenib in the treatment of melanomas expressing mutant B-RAF V600E proteins.

## Introduction

Great strides have been made over the past 10 years in the treatment of cutaneous melanoma particularly in the use of checkpoint inhibitory immunotherapy and in tumors expressing mutant B-RAF V600E, the combined use of B-RAF and MEK1/2 inhibitors (1, 2). The kinase B-RAF, along with its family member RAF-1, are the membrane proximal upstream activators of the ERK1/2 MAP kinase pathway (3, 4). In comparison to RAF-1, activating mutations of B-RAF are relatively common in a variety of malignancies, particularly in cutaneous melanoma (5, 6). To combat mutant B-RAF, specific inhibitors of the mutated B-RAF V600E protein were developed, e.g. vemurafenib, dabrafenib, encorafenib (5, 6). Subsequently, it was discovered that the use of the specific mutant B-RAF inhibitors caused activation of RAF-1 and reactivation of the ERK1/2 pathway (7, 8). Hence, the therapeutic modality of combined mutant B-RAF inhibition with MEK1/2 inhibition was developed, e.g. dabrafenib plus trametinib (9), vemurafenib and cobimetinib and encorafenib and binimetinib. However, despite an initial response of mutant B-RAF V600E cutaneous melanoma cells to the kinase inhibitory drug combination, tumor cells within 12-18 months eventually become drug-resistant and clinical progression occurs. Thus, novel alternative approaches are still required to attack drug-resistant mutant B-RAF V600E melanoma, and to increase its sensitivity to immunotherapy.

The novel cancer therapeutic GZ17-6.02 undergoing phase I evaluation in solid tumor patients. GZ17-6.02 is comprised of three natural chemicals; curcumin (10%); isovanillin (77%); and harmine (13%) (10–12). The most widely studied of these three compounds is curcumin; turmeric, the spice most associated with Indian cuisine is comprised ~95% of curcumin and curcuminoid derivatives. The safe maximal plasma concentration of commercially available lecithin liposomal curcumin, e.g. Meriva^®^, for an 800 mg ingestion is approximately 2 µM. And, our prior *in vitro* studies with curcumin as a single agent have also used the compound at a 2.0 µM final concentration. Thus, we used GZ17-6.02 for our current *in vitro* studies with the basal concentration of curcumin set at 2.0 µM.

The plant *Arum palaestinum* has been used for centuries in the Levant for the treatment of many ailments, including cancer (13). The plant *Peganum harmala* has also been used in the near-east as a medicinal herb for millennia, and also for the treatment of malignancies (14). The most bio-active chemical isolated from these plants is harmine. Studies have shown that whilst harmine has anti-proliferative effects in tumor cells, the compound appears to lack any anti-proliferative biologic effects in non-transformed cells (15–17). It has also been argued that harmine can damage DNA, inhibiting the relaxation activity of DNA topoisomerase I and II, and inhibit drug efflux pumps (15, 16). Studies in the yeast *Saccharomyces cerevisiae* have been performed to test harmine for any putative genotoxicity, mutagenicity and recombinogenic toxicity. Harmine-induced crossing-over and frameshift mutagens in yeast (17). Mutants defective in nucleotide excision repair, in error-prone repair and in recombinational repair had enhanced sensitivity to harmine, with Rad1/Rad6 double mutants demonstrating that both NER and error-prone repair are independently involved in repair of harmine-induced DNA lesions. In general agreement with these findings, harmine has also been shown to increase the numbers of micronuclei in eukaryotic cells and in the traditional Ames test, harmine-induced frameshift mutations in *S. typhimurium* strains TA97 and TA98 (18).

We have published that GZ17-6.02 killed GI tumor cells and interacted with 5-fluorouracil (5FU) to further enhance tumor cell death *in vitro* and *in vivo* (10). In GI tumor cells GZ17-6.02 activated an ATM-AMPK signaling module which was responsible for inactivation of mTORC1 and mTORC2, dephosphorylation of ULK1 S757, and increased phosphorylation of ULK1 S317 and of ATG13 S318. Autophagosome formation and autophagic flux were observed. GZ17-6.02 and 5FU caused greater ATM activation, more autophagosome formation and more killing; knock down of ULK1, Beclin1 or ATG5 suppressed the lethality of GZ17-6.02. The present studies were performed to determine whether GZ17-6.02 alone or combined with the standard of care drugs [trametinib + dabrafenib] killed cutaneous melanoma cells expressing mutant B-RAF V600E and then to define the multiple mechanisms by which cell death occurred.

## Materials and Methods

### Materials

PDX (patient derived xenograft) cutaneous B-RAF V600E melanoma isolates were sub-cultured from the patient into the flanks of NRG mice; they were supplied by Dr. Kirkwood from the University of Pittsburgh cell bank: TPF-8-196 (MEL1), TPF-12-293 (MEL2), TPF-12-510 (MEL3), TPF-12-198 (MEL4), TPF-12-542 (MEL5), TPF-11-1081 (MEL6). Dabrafenib and trametinib were purchased from Selleckchem (Houston, TX). All Materials were obtained as described in the references (19–24). Trypsin-EDTA, DMEM, RPMI, penicillin-streptomycin were purchased from GIBCOBRL (GIBCOBRL Life Technologies, Grand Island, NY). Other reagents and performance of experimental procedures were as described (19–24). Antibodies used: AIF (5318), BAX (5023), BAK (12105), BAD (9239), BIM (2933), BAK1 (12105), Beclin1 (3495), cathepsin B (31718), CD95 (8023), FADD (2782), eIF2α (5324), P-eIF2α S51 (3398), ULK-1 (8054), P-ULK-1 S757 (14202), P-AMPK S51 (2535), AMPKα (2532), P-ATM S1981 (13050), ATM (2873), ATG5 (12994), mTOR (2983), P-mTOR S2448 (5536), P-mTOR S2481 (2974), ATG13 (13468), MCL-1 (94296), BCL-XL (2764), P-AKT T308 (13038), P-ERK1/2 (5726), P-STAT3 Y705 (9145), P-p65 S536 (3033), p62 (23214), LAMP2 (49067) all from Cell Signaling Technology (Danvers, MA); P-ULK-1 S317 (3803a) was from Abgent; P-ATG13 S318 (19127) from Novus Biologicals. Anti-PD-L1, PD-L2 and MHCA antibodies were from ABCAM (Cambridge, UK). The ODC antibody was purchased from Santa Cruz Biotechnology (Dallas, TX). Specific multiple independent siRNAs to knock down the expression of CD95, FADD, Beclin1, ATG5 and eIF2α, and scramble control, were purchased from Qiagen (Hilden Germany). Control studies were presented showing on-target specificity of our siRNAs, primary antibodies and our phospho-specific antibodies to detect both total protein levels and phosphorylated levels of proteins (10–12, 19–24).

### Methods

All bench-side Methods used in this manuscript have been performed and described in the peer-reviewed references (10–12, 19–24). All cell lines were cultured at 37°C (5% (v/v CO2) *in vitro* using RPMI supplemented with dialyzed 5% (v/v) fetal calf serum and 1% (v/v) Non-essential amino acids. Drugs are dissolved in DMSO to make 10 mM stock solutions. The stock solution is diluted to the desired concentration in the media that the cells being investigated grow in. We ensure that the concentration of DMSO is never more than 0.1% (v/v) in the final dilution that is added to cells, to avoid solvent effects. Cells were not cultured in reduced serum media during any study in this manuscript.

### Assessments of Protein Expression and Protein Phosphorylation

Multi-channel fluorescence HCS microscopes perform true in-cell western blotting. Three independent cultures derived from three thawed vials of cells of a tumor were sub-cultured into individual 96-well plates. Twenty-four hours after plating, the cells are transfected with a control plasmid or a control siRNA, or with an empty vector plasmid or with plasmids to express various proteins. After another 24 hours, the cells are ready for drug exposure(s). At various time-points after the initiation of drug exposure, cells are fixed in place using paraformaldehyde and using Triton X100 for permeabilization. Standard immunofluorescent blocking procedures are employed, followed by incubation of different wells with a variety of validated primary antibodies and subsequently validated fluorescent-tagged secondary antibodies are added to each well. The microscope determines the background fluorescence in the well and in parallel randomly determines the mean fluorescent intensity of 100 cells per well. Of note for scientific rigor is that the operator does not personally manipulate the microscope to examine specific cells; the entire fluorescent accrual method is independent of the operator.

For co-localization studies, three to four images of cells stained in the red and green fluorescence channels are taken for each treatment/transfection/condition. Images are approximately 4 MB sized files. Images are merged in Adobe Photoshop CS5 and the image intensity and contrast is then post-hoc altered in an identical fashion inclusive for each group of images/treatments/conditions, so that the image with the weakest intensity is still visible to the naked eye for publication purposes but also that the image with the highest intensity is still within the dynamic range, i.e. not over-saturated.

### Detection of Cell Death by Trypan Blue Assay

Cells were treated with vehicle control or with drugs alone or in combination. At the indicated time points cells were harvested by trypsinization and centrifugation. Cell pellets were resuspended in PBS and mixed with trypan blue agent. Viability was determined microscopically using a hemocytometer. Five hundred cells from randomly chosen fields were counted and the number of dead cells was counted and expressed as a percentage of the total number of cells counted.

## Transfection of Cells With siRNA or With Plasmids

### For Plasmids

Cells were plated and 24h after plating, transfected. Plasmids to express FLIP-s (16016), BCL-XL (46972), dominant negative caspase 9 (11819), activated AKT (11547), activated mTOR (19994) and activated MEK1 EE (31880) were used throughout the study (Addgene, Waltham, MA). Plasmids expressing a specific mRNA or appropriate empty vector control plasmid (CMV) DNA was diluted in 50 µl serum-free and antibiotic-free medium (1 portion for each sample). Concurrently, 2 µl Lipofectamine 2000 (Invitrogen), was diluted into 50 µl of serum-free and antibiotic-free medium (1 portion for each sample). Diluted DNA was added to the diluted Lipofectamine 2000 for each sample and incubated at room temperature for 30 min. This mixture was added to each well/dish of cells containing 100 µl serum-free and antibiotic-free medium for a total volume of 300 µl, and the cells were incubated for 4 h at 37°C. An equal volume of 2x serum containing medium was then added to each well. Cells were incubated for 24h, then treated with drugs.

### Transfection for siRNA

Cells from a fresh culture growing in log phase as described above, and 24h after plating transfected. Prior to transfection, the medium was aspirated, and serum-free medium was added to each plate. For transfection, 10 nM of the annealed siRNA or the negative control (a “scrambled” sequence with no significant homology to any known gene sequences from mouse, rat or human cell lines) were used. Ten nM siRNA (scrambled or experimental) was diluted in serum-free media. Four ml Hiperfect (Qiagen) was added to this mixture and the solution was mixed by pipetting up and down several times. This solution was incubated at room temp for 10 min, then added dropwise to each dish. The medium in each dish was swirled gently to mix, then incubated at 37°C for 2h. Serum-containing medium was added to each plate, and cells were incubated at 37°C for 24h before then treated with drugs (0-24h).

### Assessments of Autophagosome and Autolysosome Levels

Cells were transfected with a plasmid to express LC3-GFP-RFP (Addgene, Watertown MA). Twenty-four h after transfection, cells are treated with vehicle control or GZ17-6.02, [dabrafenib + trametinib] or the drug combination. Cells were imaged at 60X magnification 4 h and 8 h after drug exposure and the mean number of GFP+ and RFP+ puncta per cell determined from >50 randomly selected cells per condition.

### Data Analysis

Comparison of the effects of various treatments was using one-way ANOVA for normalcy followed by a two tailed Student’s t-test. Differences with a p-value of < 0.05 were considered statistically significant. Experiments are the means of multiple individual data points per experiment from 3 independent experiments (± SD).

## Results

**Figure 1:**
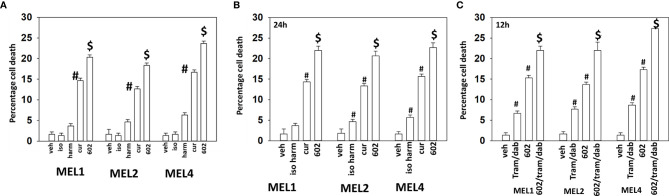
GZ17-6.02 interacts with [trametinib + dabrafenib] to kill cutaneous melanoma cells expressing B-RAF V600E. **(A)** Melanoma cells were treated with vehicle control, isovanillin (37.2 µM), harmine (4.5 µM), curcumin (2 µM) or GZ17-6.02 [curcumin (2 µM) + isovanillin (37.2 µM) + harmine (4.5 µM)] for 24h. Cells were isolated and viability determined by trypan blue exclusion (n = 3 +/- SD). ^#^p < 0.05 greater than vehicle control; ^$^p < 0.05 greater than value in cells treated with curcumin alone. **(B)** Melanoma cells were treated with vehicle control, [isovanillin (37.2 µM) + harmine (4.5 µM)], curcumin (2 µM) or GZ17-6.02 [curcumin (2 µM) + isovanillin (37.2 µM) + harmine (4.5 µM)] for 24h. Cells were isolated and viability determined by trypan blue exclusion (n = 3 +/- SD). ^#^p < 0.05 greater than vehicle control; ^$^p < 0.05 greater than value in cells treated with curcumin alone. **(C)** Melanoma cells were treated with vehicle control, GZ17-6.02 [curcumin (2 µM) + isovanillin (37.2 µM) + harmine (4.5 µM)], [trametinib (Tram, 2 µM) + dabrafenib (dab, 2 µM)] or the drugs in combination for 12h. Cells were isolated and viability determined by trypan blue exclusion (n = 3 +/- SD). ^#^p < 0.05 greater than vehicle control; ^$^p < 0.05 greater than value in cells treated with GZ17-6.02 alone.

Our initial studies using GZ17-6.02 in cutaneous mutant B-RAF V600E melanoma cells defined the relative importance of each component in the three-compound isovanillin/harmine/curcumin mixture and whether GZ17-6.02 interacted with [trametinib + dabrafenib] to cause additional cell killing. Treatment of cells with isovanillin did not cause appreciable tumor cell killing and the ability of harmine to cause cell death was modest, however curcumin did cause significant levels of cell killing (**Figure 1A**). GZ17-6.02, however, caused significantly more killing than curcumin alone or the additive lethal effects of harmine and curcumin. As a single agent curcumin caused significant levels of melanoma cell death, whereas the impact of [isovanillin + harmine] on viability was again modest (**Figure 1B**). [Isovanillin + harmine] significantly further enhanced curcumin lethality in a greater than additive fashion. Surprisingly, using supra-physiologic concentrations of [trametinib + dabrafenib], low concentrations of GZ17-6.02 were more efficacious at killing melanoma cells (**Figure 1C**). The kinase inhibitors and GZ17-6.02 interacted in an additive fashion to kill.

**Figure 2:**
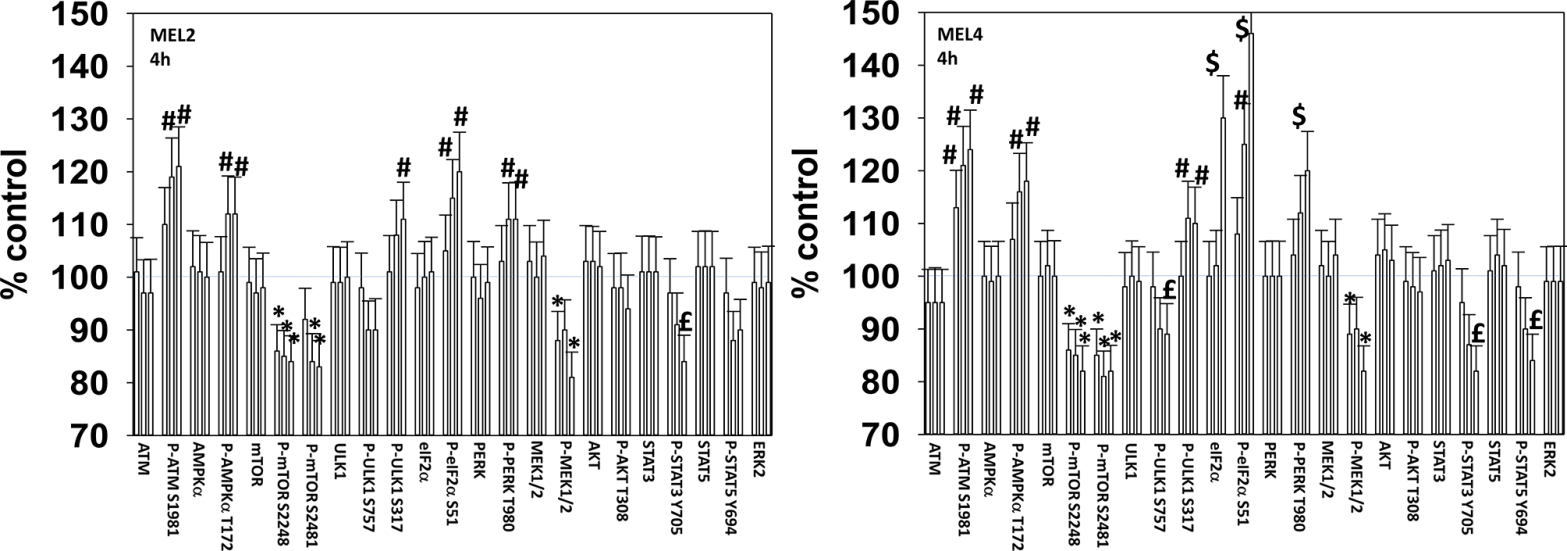
****GZ17-6.02 and [trametinib + dabrafenib] interact to increase endoplasmic reticulum stress signaling and inactivate STAT transcriptionfactors. Melanoma cells were treated with vehicle control, GZ17-6.02 [curcumin (2 µM) + isovanillin (37.2 µM) + harmine (4.5 µM)], [trametinib (2 µM) + dabrafenib (2 µM)] or the drugs in combination for 4h (the three bars per protein from left to right are trametinib/dabrafenib, GZ17-6.02 and the drugs combined). Cells were fixed in place and in-cell immunostaining performed to determine the expression and the phosphorylation of the indicated proteins (n = 3 +/-SD) *p < 0.05 less than vehicle control; £ p < 0.05 less than either drug as a single agent; ^#^p < 0.05 greater than vehicle control; ^$^p < 0.05 greater than either drug as a single agent.

We next defined over a time course the impact of GZ17-6.02, the kinase inhibitors, and the combination on cell signaling parameters in the MEL4 isolate and in the MEL2 isolate that is vemurafenib resistant. For each group of three bars, left to right, the treatments are [dabrafenib + trametinib], GZ17-6.02 and the agents combined. In the MEL4 isolate, after 4h, the kinase inhibitors and GZ17-6.02 interacted to reduce the phosphorylation of ULK1 S757, STAT3 Y705, STAT5 Y694 and to increase the phosphorylation of PERK T980 and eIF2α S51 (**Figure 2**). In the MEL2 isolate, the combination interacted to reduce STAT3 Y705, but not STAT5 Y694, and to increase ULK1 S317 phosphorylation, but generally the effects of the individual treatments on signaling were of a lower amplitude than observed in MEL4 cells.

**Figure 3:**
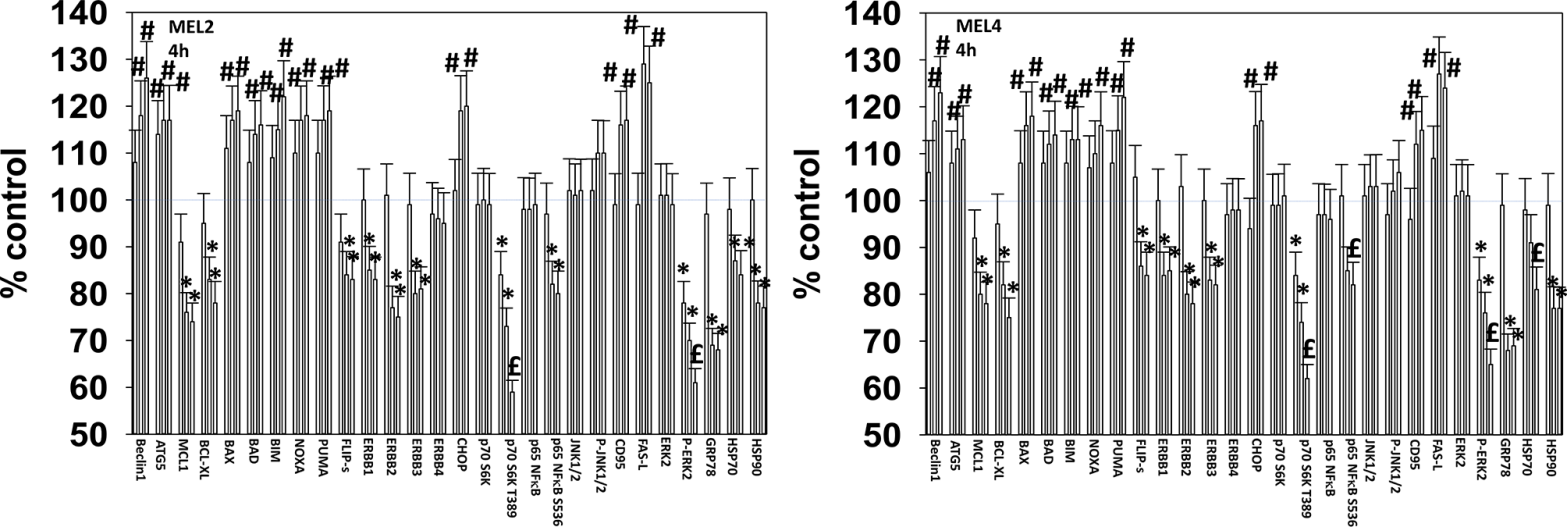
GZ17-6.02 and [trametinib + dabrafenib] interact to inactivate ERK1/2 and p70 S6K.**** Melanoma cells were treated with vehicle control, GZ17-6.02 [curcumin (2 µM) + isovanillin (37.2 µM) + harmine (4.5 µM)], [trametinib (2 µM) + dabrafenib (2 µM)] or the drugs in combination for 4h. (the three bars per protein from left to right are trametinib/dabrafenib, GZ17-6.02 and the drugs combined). Cells were fixed in place and in-cell immunostaining performed to determine the expression and the phosphorylation of the indicated proteins (n = 3 +/-SD) *p < 0.05 less than vehicle control; ^£^p < 0.05 less than either drug as a single agent; ^#^p < 0.05 greater than vehicle control; ^$^p < 0.05 greater than either drug as a single agent.

**Figure 4:**
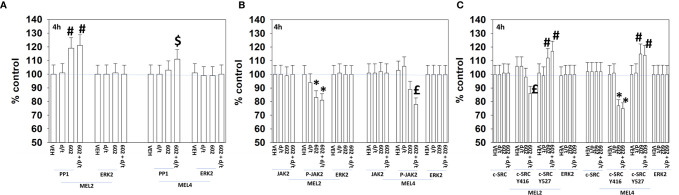
GZ17-6.02 and [trametinib + dabrafenib] can interact to inactivate c-SRC and JAK2 and increase expression of protein serine/threonine phosphatase 1. **(A–C)** Melanoma cells were treated with vehicle control, GZ17-6.02 [curcumin (2 µM) + isovanillin (37.2 µM) + harmine (4.5 µM)], [trametinib (t, 2 µM) + dabrafenib (d, 2 µM)] or the drugs in combination for 4h. Cells were fixed in place and in-cell immunostaining performed to determine the expression and the phosphorylation of the indicated proteins (n = 3 +/-SD) *p < 0.05 less than vehicle control; ^£^p < 0.05 less than either drug as a single agent; ^#^p < 0.05 greater than vehicle control; ^$^p < 0.05 greater than either drug as a single agent.

For other signaling parameters, however, MEL4 and MEL2 exhibited similar responses (**Figure 3**). In both isolates combination of the agents lead to a greater dephosphorylation of p70 S6K T389 and of ERK1/2 T185/Y187, and reduced HSP70 expression. As a single agent in both isolates, GZ17-6.02 reduced the expression of FLIP-s, MCL1 and BCL-XL and increased the levels of BAX, BAD, BIM, Noxa, Puma, CHOP, CD95, FAS-L, GRP78, HSP90, Beclin1 and ATG5. In MEL4 cells, GZ17-6.02 and [trametinib + dabrafenib] combined to increase the expression of protein Ser/Thr phosphatase 1 and decrease the phosphorylation of JAK2 Y1007 (**Figures 4A, B** **)**. In MEL2 cells as a single agent, and unaltered by the kinase inhibitors, GZ17-6.02 enhanced PP1 levels and decreased JAK2 phosphorylation. GZ17-6.02 decreased c-SRC Y416 and increased c-SRC Y527 phosphorylation, which is indicative that c-SRC was being inactivated (**Figure 4C**).

**Figure 5:**
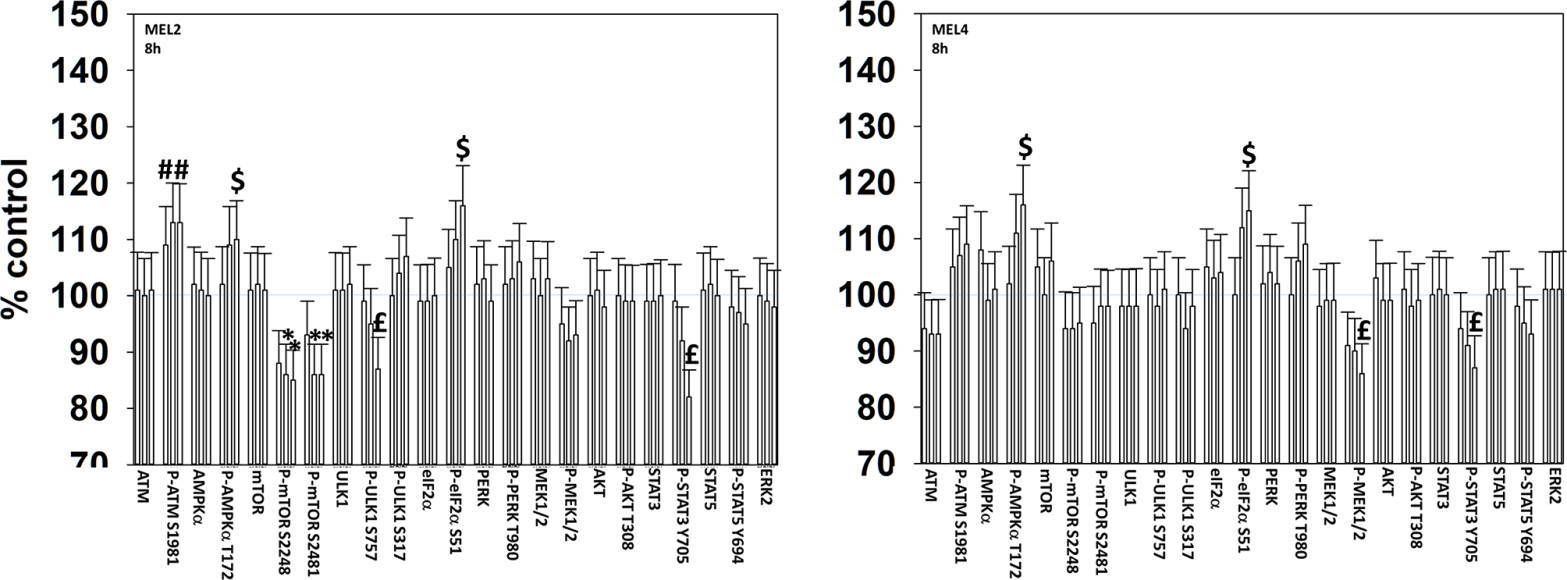
Exposure to [GZ17-6.02 + trametinib + dabrafenib] maintains elevated endoplasmic reticulum stress signaling and inactivation of STAT transcription factors.**** Melanoma cells were treated with vehicle control, GZ17-6.02 [curcumin (2 µM) + isovanillin (37.2 µM) + harmine (4.5 µM)], [trametinib (2 µM) + dabrafenib (2 µM)] or the drugs in combination for 8h. (the three bars per protein from left to right are trametinib/dabrafenib, GZ17-6.02 and the drugs combined). Cells were fixed in place and in-cell immunostaining performed to determine the expression and the phosphorylation of the indicated proteins (n = 3 +/-SD) *p < 0.05 less than vehicle control; ^£^p < 0.05 less than either drug as a single agent; ^#^p < 0.05 greater than vehicle control; ^$^p < 0.05 greater than either drug as a single agent.

**Figure 6:**
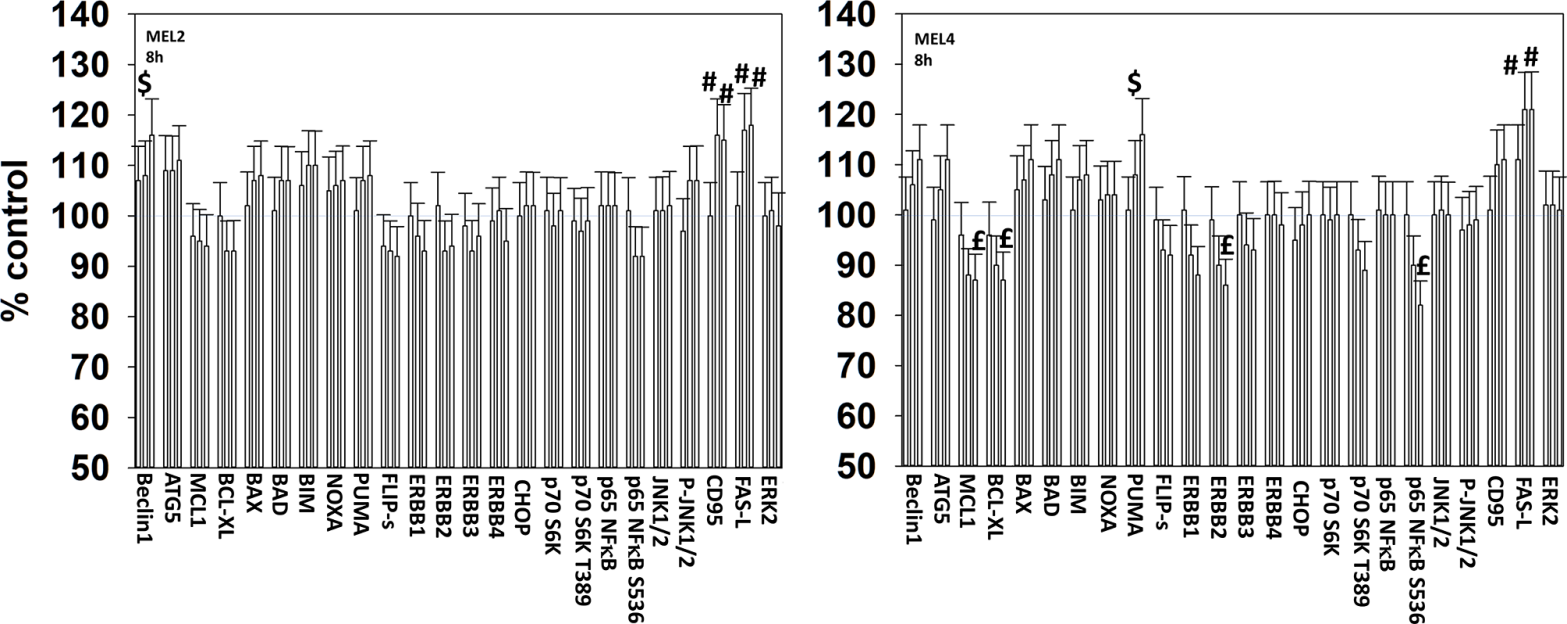
Exposure to [GZ17-6.02 + trametinib + dabrafenib] has the potential to increase the expression of ATG5 or reduce MCL1 and BCL-XL levels.**** Melanoma cells were treated with vehicle control, GZ17-6.02 [curcumin (2 µM) + isovanillin (37.2 µM) + harmine (4.5 µM)], [trametinib (2 µM) + dabrafenib (2 µM)] or the drugs in combination for 8h. (the three bars per protein from left to right are trametinib/dabrafenib, GZ17-6.02 and the drugs combined). Cells were fixed in place and in-cell immunostaining performed to determine the expression and the phosphorylation of the indicated proteins (n = 3 +/-SD) *p < 0.05 less than vehicle control; ^£^p < 0.05 less than either drug as a single agent; ^#^p < 0.05 greater than vehicle control; ^$^p < 0.05 greater than either drug as a single agent.

We next determined changes in cell signaling parameters, 8h after drug exposure. In both MEL2 and MEL4 cells, the drug combination maintained the phosphorylation of AMPKα T172 and eIF2α S51 and the dephosphorylation of STAT3 Y705 (**Figure 5**). Although the drugs combined to reduce MEK1/2 phosphorylation in MEL4 cells, this effect was not observed in the vemurafenib resistant MEL2 isolate. Similar observations were made for MCL1 and BCL-XL (**Figure 6**). The MEL4 isolate the expression of FAS-L was elevated and in the MEL2 isolate, expression of CD95 also remained higher. The trend for toxic BH3 domain protein expression in toto in both isolates was that their expression was increased above basal levels, and the levels of Puma remained high in MEL4 cells.

**Figure 7:**
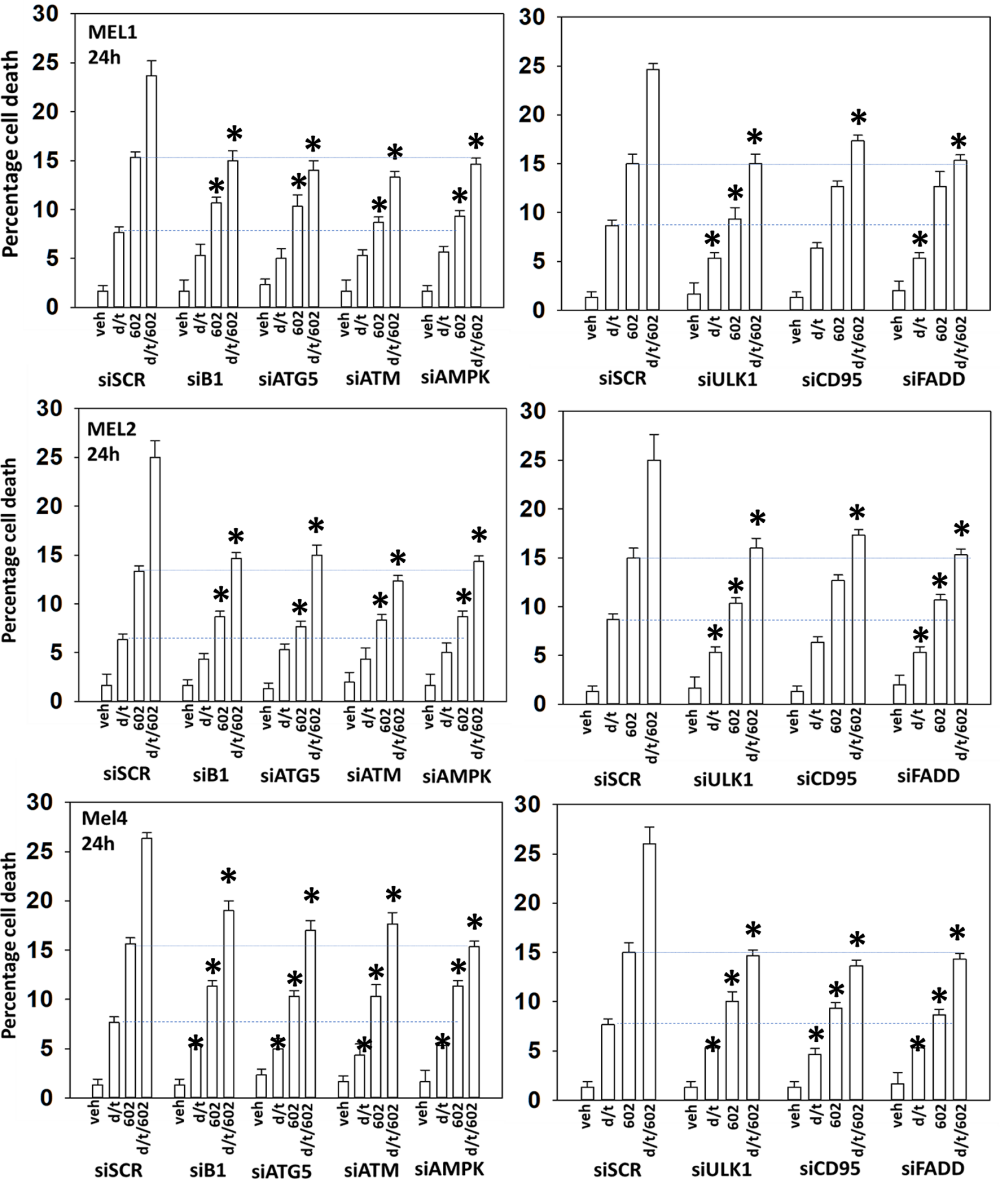
Enhanced tumor cell killing by [GZ17-6.02 + trametinib + dabrafenib] requires autophagosome formation and CD95 death receptor signaling.**** Cells were transfected with a scrambled siRNA (siSCR) or with validated siRNA molecules to knock down the expression of the indicated proteins. Twenty-four h later, cells were treated with vehicle control, GZ17-6.02 [curcumin (2 µM) + isovanillin (37.2 µM) + harmine (4.5 µM)], [trametinib (t, 2 µM) + dabrafenib (d, 2 µM)] or the drugs in combination for 24h. Cells were isolated and viability determined by trypan blue exclusion (n = 3 +/- SD). *p < 0.05 less than corresponding value in siSCR transfected cells.

**Figure 8:**
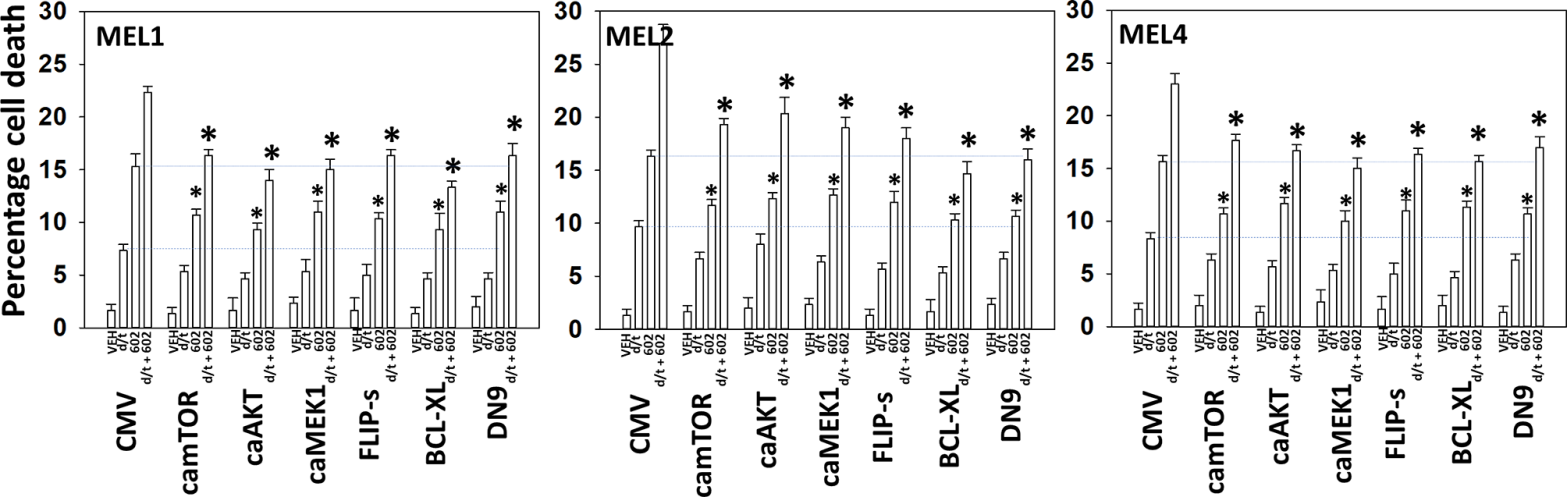
Enhanced tumor cell killing by [GZ17-6.02 + trametinib + dabrafenib] requires mitochondrial dysfunction.**** Cells were transfected with an empty vector plasmid (CMV) or with plasmids to express the indicated proteins. Twenty-four h later, cells were treated with vehicle control, GZ17-6.02 [curcumin (2 µM) + isovanillin (37.2 µM) + harmine (4.5 µM)], [trametinib (t, 2 µM) + dabrafenib (d, 2 µM)] or the drugs in combination for 24h. Cells were isolated and viability determined by trypan blue exclusion (n = 3 +/- SD). *p < 0.05 less than corresponding value in siSCR transfected cells.

We next determined a pathway analysis to determine the mechanisms of cell killing. Prior studies in other cell types have shown GZ17-6.02 killing through mechanisms using the death receptor CD95 and autophagosome formation (10–12). We also previously demonstrated using other agents that activation of ATM can lead to activation of the AMPK followed by mTOR inactivation, ULK1 activation, ATG13 S318 phosphorylation and autophagosome formation (22). Knock down of ULK1, ATG5 or Beclin1 significantly reduced tumor cell killing by the drug combination (**Figure 7**). Similar data were obtained knocking down ATM or AMPKα or knocking down CD95 or FADD. In further agreement with the CD95/FADD knock down data, over-expression of the caspase 8/10 inhibitor FLIP-s also suppressed cell death (**Figure 8**). The drug treatments individually and combined reduced the activities of mTOR, AKT and MEK1/2-ERK1/2. Expression of mutant active forms of mTOR, AKT or MEK1 significantly reduced drug combination lethality. Downstream of death receptors and autophagy is the mitochondrion. The drug combination reduced expression of MCL1 and BCL-XL and over-expression of the mitochondrial protective protein BCL-XL reduced cell death (**Figure 8**).

**Figure 9:**
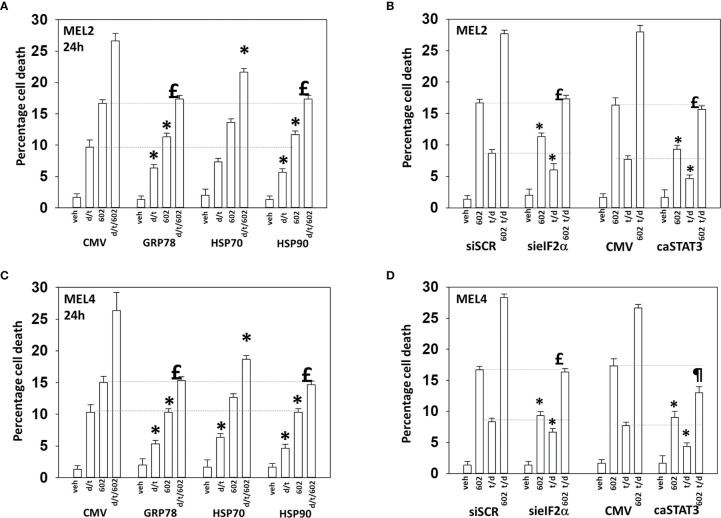
Over-expression of GRP78 or HSP90, to a greater extent than HSP70, reduces drug lethality in melanoma cells. **(A)** and **(B)** Melanoma cells were transfected with an empty vector plasmid (CMV) or with plasmids to express the chaperones GRP78, HSP70 or HSP90. Twenty-four h later, cells were treated with vehicle control, GZ17-6.02 [curcumin (2 µM) + isovanillin (37.2 µM) + harmine (4.5 µM)], [trametinib (t, 2 µM) + dabrafenib (d, 2 µM)] or the drugs in combination for 24h. Cells were isolated and viability determined by trypan blue exclusion (n = 3 +/- SD). *p < 0.05 less than corresponding value in CMV transfected cells; £ p > 0.05 comparing d/t/602 value to 602 alone value in CMV cells. **(C)** and **(D)** Cells were transfected with a scrambled siRNA or with a validated siRNA molecule to knock down the expression of eIF2α. Cells were transfected with an empty vector plasmid (CMV) or with a plasmid to express activated STAT3. Twenty-four h later, cells were treated with vehicle control, GZ17-6.02 [curcumin (2 µM) + isovanillin (37.2 µM) + harmine (4.5 µM)], [trametinib (t, 2 µM) + dabrafenib (d, 2 µM)] or the drugs in combination for 24h. Cells were isolated and viability determined by trypan blue exclusion (n = 3 +/- SD). *p < 0.05 less than corresponding value in transfected cells; ^£^p > 0.05 comparing d/t/602 value to 602 alone value in their corresponding transfected cells; ^¶^p < 0.05 less than 602 single agent value in CMV transfected cells.

Endoplasmic reticulum stress, as manifested by eIF2α S51 phosphorylation, was maintained for at least 8h after drug exposure. Over-expression of GRP78, which inhibits PERK signaling to eIF2α, HSP90 or HSP70 significantly reduced the lethality of the agents individually and combined (**Figures 9A, B**). Surprisingly, although the agents interacted to further reduce HSP70 expression, over-expression of HSP70 was less protective when compared to GRP78 or HSP90. Knock down of eIF2α or expression of activated STAT3 suppressed tumor cell killing reducing the lethality of the three-drug combination to that of GZ17-6.02 alone (**Figures 9C, D**). Collectively the data in **Figures 7**–**9** indicates that GZ17-6.02 and [trametinib + dabrafenib] interact to kill PDX isolates of cutaneous mutant B-RAF V600E expressing cells *via* activation of death receptor signaling and autophagy resulting in mitochondrial dysfunction leading to apoptotic and non-apoptotic forms of cell killing.

**Figure 10:**
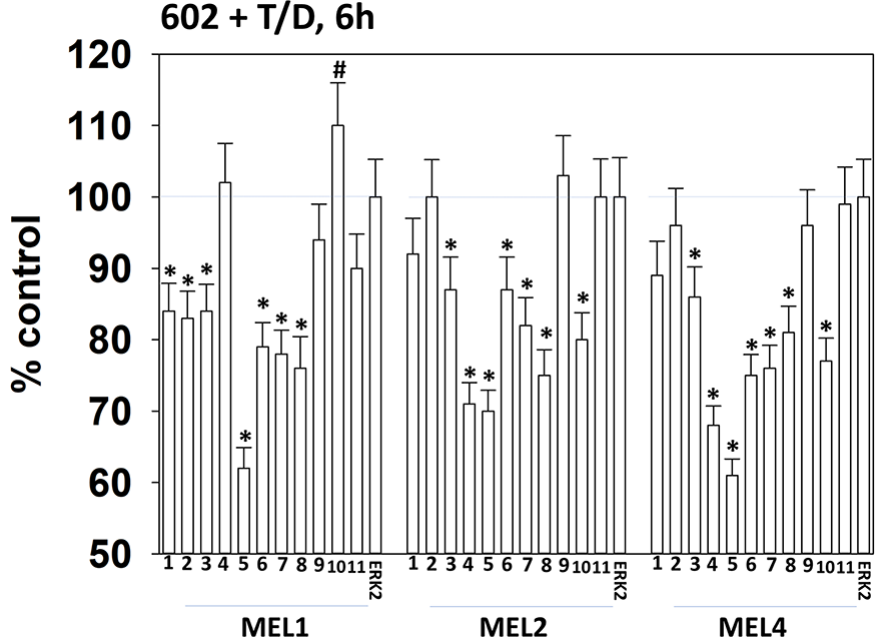
Exposure of cells to [GZ17-6.02 + trametinib + dabrafenib] reduces the expression of multiple HDAC proteins.**** Melanoma cells were treated with vehicle control or [GZ17-6.02 [curcumin (2 µM) + isovanillin (37.2 µM) + harmine (4.5 µM)] + trametinib (t, 2 µM) + dabrafenib (d, 2 µM)] for 6h. Cells were fixed in place and in-cell immunostaining performed to determine the expression of HDACs1-11 and total ERK2 as a loading control. (n = 3 +/-SD) *p < 0.05 less than vehicle control value. ^#^Greater than vehicle control.

Drugs that induce autophagosome formation and autophagic flux can reduce the protein levels of histone deacetylase proteins. The combination of GZ17-6.02 with [trametinib + dabrafenib] reduced the expression of multiple HDAC proteins. The HDACs whose expression was reduced in all tested isolates were HDAC3, HDAC5, HDAC6, HDAC7 and HDAC8 (**Figure 10**). The expression of HDAC10 was reduced in MEL2 and MEL4 cells but was modestly enhanced in MEL1 cells.

**Figure 11:**
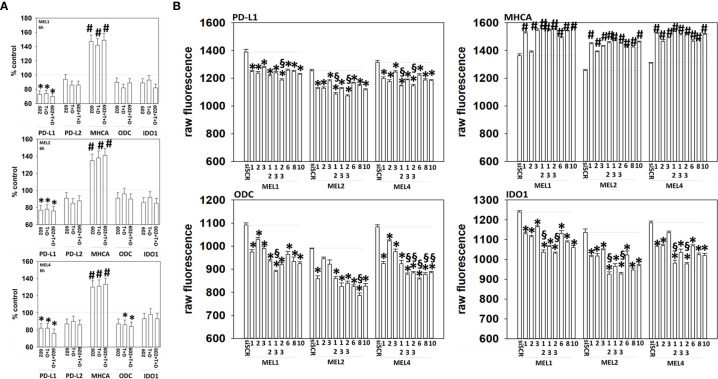
Genetic manipulation of HDAC expression results in reduced expression of PD-L1, ODC and IDO1 and elevated levels of MHCA. **(A)** Melanoma cells were treated with vehicle control, GZ17-6.02 [curcumin (2 µM) + isovanillin (37.2 µM) + harmine (4.5 µM)], [trametinib (T, 2 µM) + dabrafenib (D, 2 µM)] or the drugs in combination for 6h. Cells were fixed in place and in-cell immunostaining performed to determine the expression of the indicated proteins and total ERK2 as a loading control. Bar graphs are corrected for the total loading of ERK2. (n = 3 +/-SD) *p < 0.05 less than vehicle control; ^#^p < 0.05 greater than vehicle control. **(B)** Melanoma cells were transfected with an scrambled siRNA (siSCR) or with siRNA molecules to knock down the expression of HDAC proteins, as presented in the Figure. Twenty-four h later, cells were fixed in place and in-cell immuno-staining performed to define the expression of PD-L1, MHCA, ODC, IDO1 and the total expression of ERK2 as a loading control. The values presented in the bar graphs are corrected for total ERK2 levels under each condition. A representative study in triplicate from three independent experiments. (+/-SD) ^#^p < 0.05 greater than siSCR value; *p < 0.05 less than siSCR value. ^$^Less than individual knock down of an HDAC protein.

Melanoma cells expressing mutant B-RAF V600E can be efficaciously treated with checkpoint inhibitory antibodies. Treatment of cells with GZ17-6.02, [trametinib + dabrafenib] or the agents in combination enhanced the expression of MHCA and decreased the levels of PD-L1, which would predict for a greater anti-tumor immunotherapy response *in vivo* (**Figure 11A**). We then determined whether molecular manipulation of HDAC protein levels could alter the expression of PD-L1, MHCA, ODC and IDO1. Knock down of all HDACs tested to varying extents reduced the expression of PD-L1 with knock down of [HDAC2 + HDAC3] in all isolates and of [HDAC1 and HDAC2] in two isolates being most efficacious (**Figure 11B**, §). Knock down of HDACs almost uniformly increased MHCA levels. The ability of HDAC knock down to alter ODC expression was variable based on the isolate tested; in MEL1 knock down of [HDAC1 + HDAC3]; in MEL2 HDAC8; in MEL4 [HDAC1 + HDAC3], [HDAC2 + HDAC3], HDAC6, HDAC8 or HDAC10. In all isolates, knock down of either [HDAC1 + HDAC2] or [HDAC2 + HDAC3] was most efficacious at reducing IDO1 expression.

## Discussion

**Figure 12:**
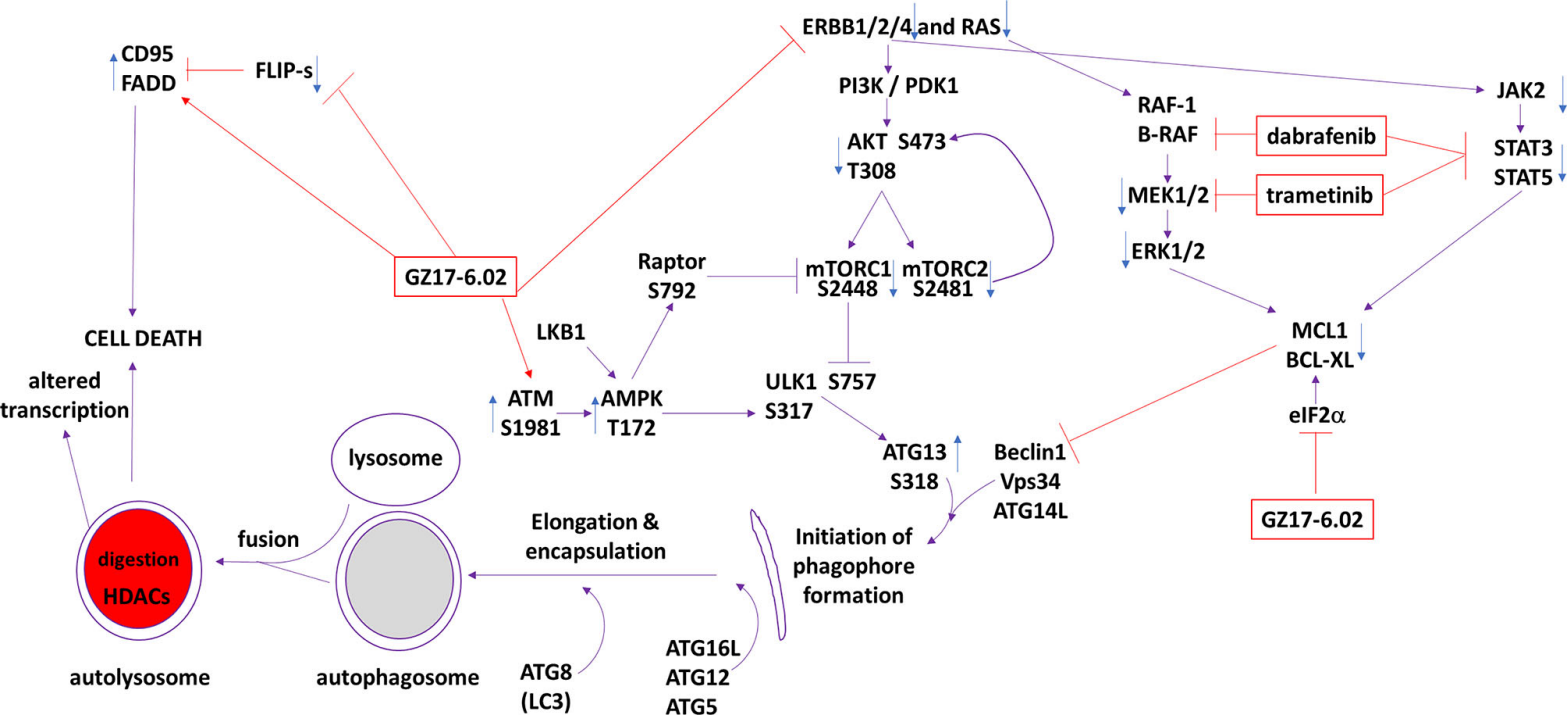
The molecular mechanisms by which GZ17-6.02 interacts with [trametinib + dabrafenib] to kill cutaneous melanoma cells that express B-RAF V600E.**** In MEL2 and MEL4 cells GZ17-6.02 initially interacted after a 4h exposure with [trametinib + dabrafenib] to cause inactivation of STAT3, p70 S6K and ERK1/2. In MEL4 cells they also combined to inactivate JAK2, STAT5, eIF2α, NFκB and reduced HSP70 levels. In MEL2 cells they also combined to inactivate c-SRC. Eight h after exposure the drugs interacted to maintain phosphorylation of the AMPK and eIF2α, and to decrease the phosphorylation of MEK1/2 and STAT3. The levels of CD95 in both isolates remained elevated at the 8h time-point. Molecular studies demonstrated that cells lacking ATM-AMPK signaling, lacking the ability to form autophagosomes or activate CD95 death receptor signaling were less able to undergo cell death processes. Over-expression of BCL-XL or dominant negative caspase 9, expression of activated STAT3 or knock down of eIF2α was more effective at suppressing cell killing than over-expression of FLIP-s or activated forms of mTOR, AKT or MEK1.

The present studies were performed to determine whether GZ17-6.02 at low concentrations could kill cutaneous melanoma cells that express the B-RAF V600E mutation and whether it interacted with the standard of care therapy, [trametinib + dabrafenib], to cause higher levels of tumor cell killing. In an additive fashion, regardless of vemurafenib resistance in MEL2 cells, GZ17-6.02 and [trametinib + dabrafenib] interacted to kill B-RAF V600E mutated melanoma cells. The principle mechanisms by which the drug combination killed were *via* death receptor activation, autophagosome formation and mitochondrial dysfunction. Expression of dominant negative caspase 9 only partially protected cells arguing that downstream of the mitochondrion, killing occurred through both apoptotic and non-apoptotic mechanisms. Enhanced endoplasmic reticulum stress signaling from PERK-eIF2α and reduced activity within the AKT/ERK/STAT3 pathways also contributed to the overall cell killing process (**Figure 12**).

The phosphorylation of eIF2α is regulated by a complex of [protein phosphatase 1 + CHOP]. In MEL2 cells GX17-6.02 as a single agent and when combined with [trametinib + dabrafenib] increased PP1 expression whereas in MEL4 cells only the drug combination could elevate PP1 levels. The expression of CHOP was enhanced by GZ17-6.02 as a single agent in both MEL2 and MEL4 cells after 4h of exposure, and in both isolates had returned to baseline by 8h. The phosphorylation of eIF2α remained elevated 8h after exposure and our data suggests that the inability of GZ17-6.02 treated cells to dephosphorylate eIF2α is probably due to reduced CHOP expression, resulting in lower levels of eIF2α/PP1 co-localization.

In other tumor cell types, we have shown that drug combinations that elevate Beclin1 and ATG5 expression and enhance autophagosome formation can reduce the levels of multiple HDAC proteins. We discovered that the combination of [GZ17-6.02 + trametinib + dabrafenib] consistently reduced the expression of HDAC3, HDAC5, HDAC6, HDAC7 and HDAC8. HDAC5 is a class II HDAC, can associate with HDAC3 and HDAC4, and acts to repress transcription, for example the transporter GLUT4 (25, 26). Over-expression of HDAC5 in melanoma, and in other malignancies, is associated with enhanced growth and a shorter patient survival (27, 28). HDAC6 is cytosolic and regulates acetylation of HSP90 and tubulin, thus impacting signaling by proteins that are chaperoned by HSP90, e.g. ERBB1, as well as altering cellular rigidity (29). In melanoma HDAC6, *via* the tyrosine phosphatase PTPN1 and enhanced ERK1/2 activity, has been proposed to enhance proliferation (30). Our data showed that the expression of HDAC6 was reduced after drug exposure and was associated with a decrease in ERK1/2 activity, which agrees with this prior publication.

Checkpoint inhibitory immunotherapy is a standard of care therapeutic approach in cutaneous melanoma. As a single agent GZ17-6.02 reduced the expression of PD-L1 and increased the levels of Class I MHCA. Both events would be predicted to enhance the efficacy of an anti-PD1 checkpoint inhibitory antibody. Molecular manipulation of HDAC expression revealed that knock down of [HDAC2 + HDAC3] was particularly efficacious at reducing PD-L1 and IDO1 levels. Knock down of [HDAC1 + HDAC3] or HDAC8 was most effective at reducing ODC expression. Recent studies have linked curcumin-induced dephosphorylation of STAT3 Y705 to increased expression of PD-L1 and an enhanced *in vivo* efficacy of an anti-PD1 antibody (31, 32). Additional studies beyond the scope of this manuscript will be required to understand how GZ17-6.02 regulates the promoters of immunotherapy-related genes.

Many studies over the past 5 years have linked resistance to B-RAF/MEK inhibitors in B-RAF V600E mutated melanoma to correlate with evolutionary survival-induced activation of ERBB family receptors, including ERBB1 and ERBB3 (33–36). GZ17-6.02 as a single agent reduced the protein levels of ERBB1, ERBB2 and ERBB3, which may contribute to preventing the development of kinase inhibitor drug resistance. In both tested isolates the drug combination also inactivated c-SRC, JAK2, with STAT3 inactivation. Reduce STAT3 signaling can result in reduced expression of multiple cytoprotective proteins, e.g. MCL1, but also, as noted above, can result in elevated PD-L1 expression and an enhanced immunotherapy response *in vivo*. Future studies will be needed to determine whether GZ17-6.02 has anti-melanoma properties *in vivo*.

## Data Availability Statement

The original contributions presented in the study are included in the article/**Supplementary Material**. Further inquiries can be directed to the corresponding author.

## Ethics Statement

The studies involving human participants were reviewed and approved by De-identified PDX isolates from The University of Pittsburgh. The patients/participants provided their written informed consent to participate in this study.

## Author Contributions

LB performed the studies under PD, who wrote the manuscript. CW and DV provided essential advice on melanoma and developmental therapeutics and read the manuscript. JK supplied the PDX melanoma cells. All authors contributed to the article and approved the submitted version.

## Funding

PD has received funding support from Genzada Pharmaceuticals Inc. The funder was not involved in the study design, collection, analysis, interpretation of data, the writing of this article or the decision to submit it for publication.

## Conflict of Interest

PD has received funding support from Genzada Pharmaceuticals Inc. for these studies. CW is a paid officer of the company. PD and DV are Consultants and Key Scientific advisors to the company.

The remaining authors declare that the research was conducted in the absence of any commercial or financial relationships that could be construed as a potential conflict of interest.

## Abbreviations

ERK, extracellular regulated kinase; PI3K, phosphatidyl inositol 3 kinase; ca, constitutively active; dn, dominant negative; ER, endoplasmic reticulum; AIF, apoptosis inducing factor; AMPK, AMP-dependent protein kinase; mTOR, mammalian target of rapamycin; JAK, Janus Kinase; STAT, Signal Transducers and Activators of Transcription; MAPK, mitogen activated protein kinase; PTEN, phosphatase and tensin homologue on chromosome ten; ROS, reactive oxygen species; CMV, empty vector plasmid or virus; si, small interfering; SCR, scrambled; IP, immunoprecipitation; VEH, vehicle; 602, GZ17-6.02; 5FU, 5-flourouracil; Iso, isovanillin; Harm, harmine; PD-L1, programed death ligand 1; HDAC, histone deacetylase.
