# Supplementary figures and images for: GZ17-6.02 Interacts With [MEK1/2 and B-RAF Inhibitors] to Kill Melanoma Cells
Source: Front Oncol. 2021 Apr 8;11:656453. doi: 10.3389/fonc.2021.656453 (PMC8061416; doi:10.3389/fonc.2021.656453)

Supplemental Figure 1

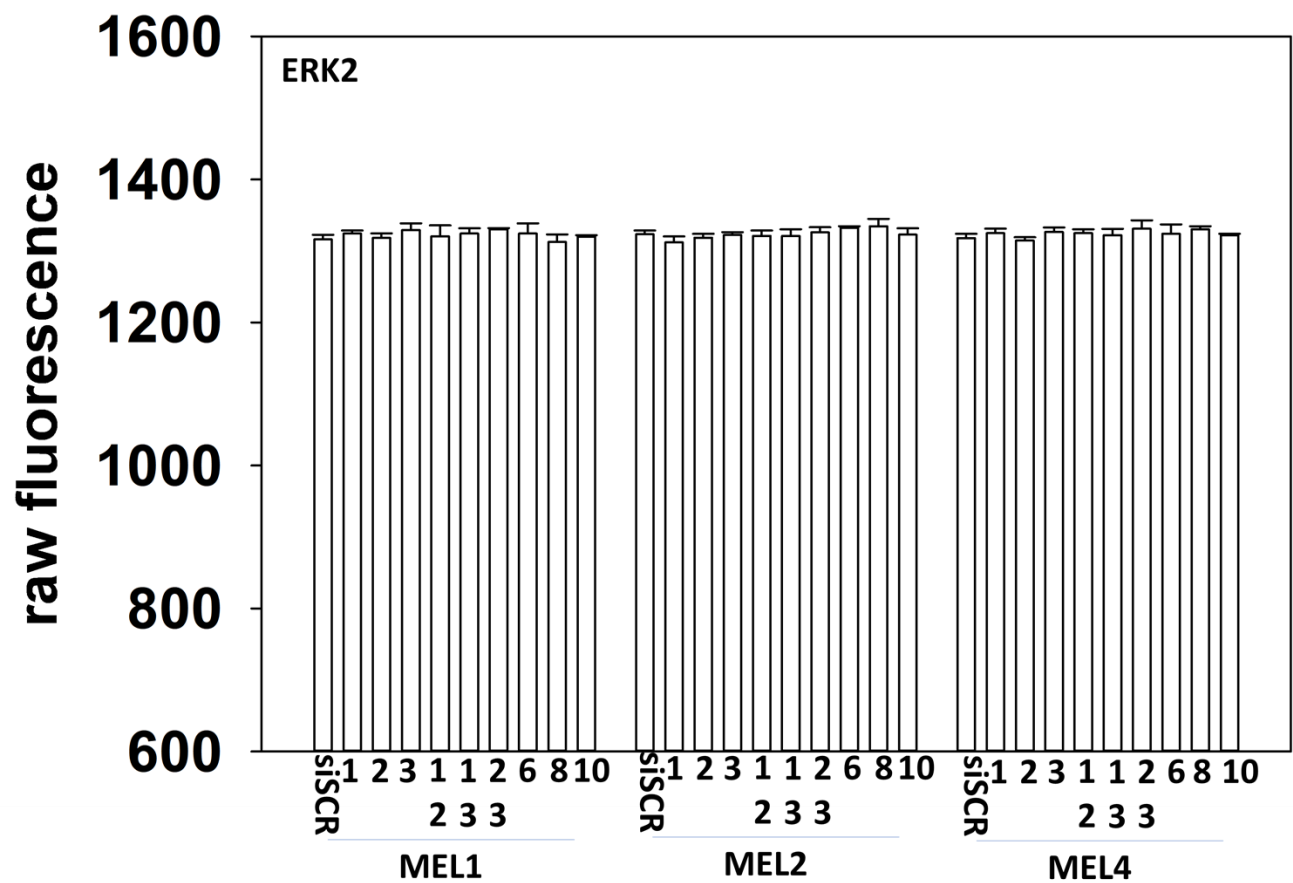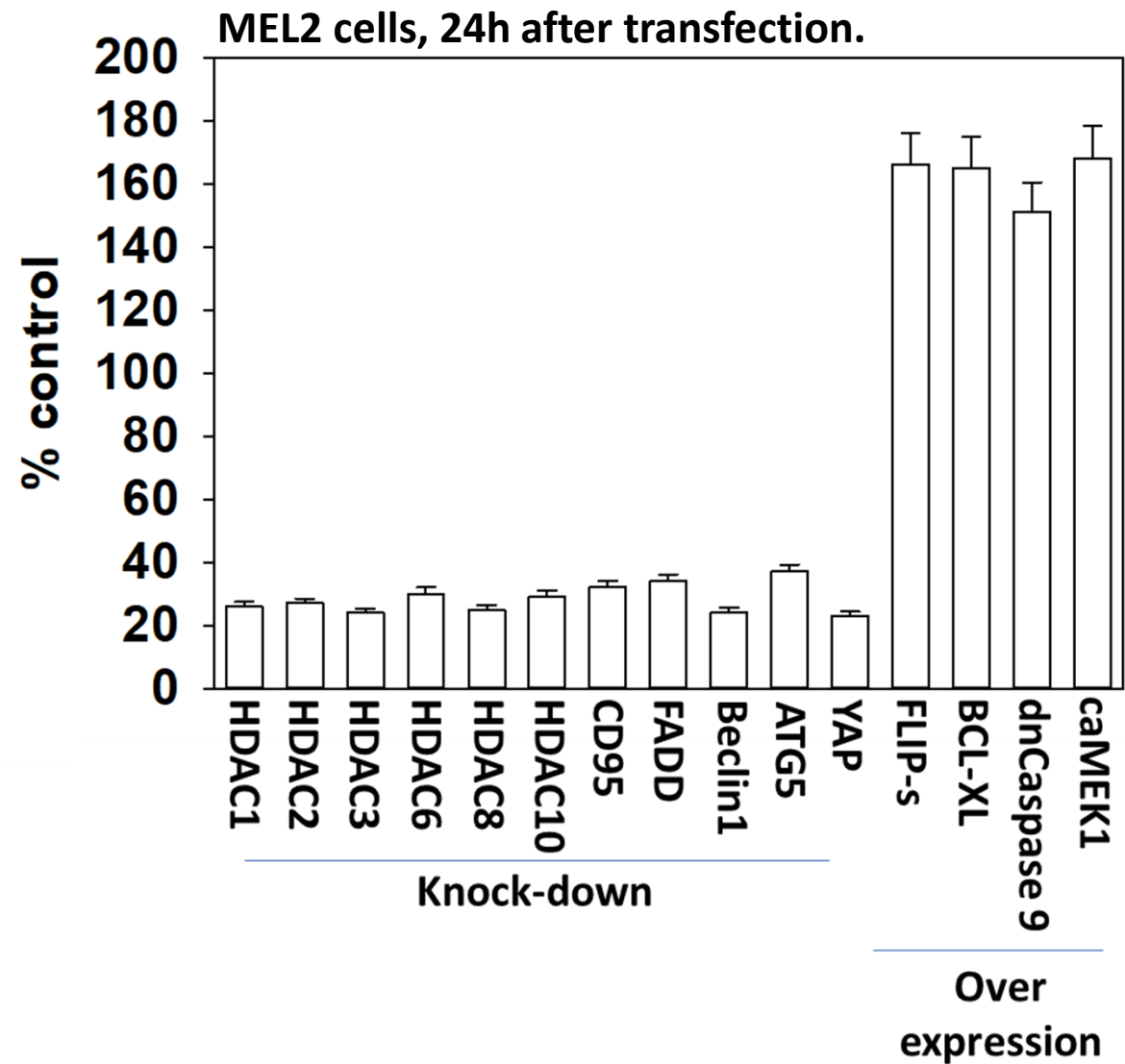

Supplement: Supplementary file 1 [file DataSheet_1.pdf]
